# Supplementary figures and images for: External validation of a claims-based model to predict left ventricular ejection fraction class in patients with heart failure
Source: PLoS One. 2021 Jun 4;16(6):e0252903. doi: 10.1371/journal.pone.0252903 (PMC8177622; doi:10.1371/journal.pone.0252903)

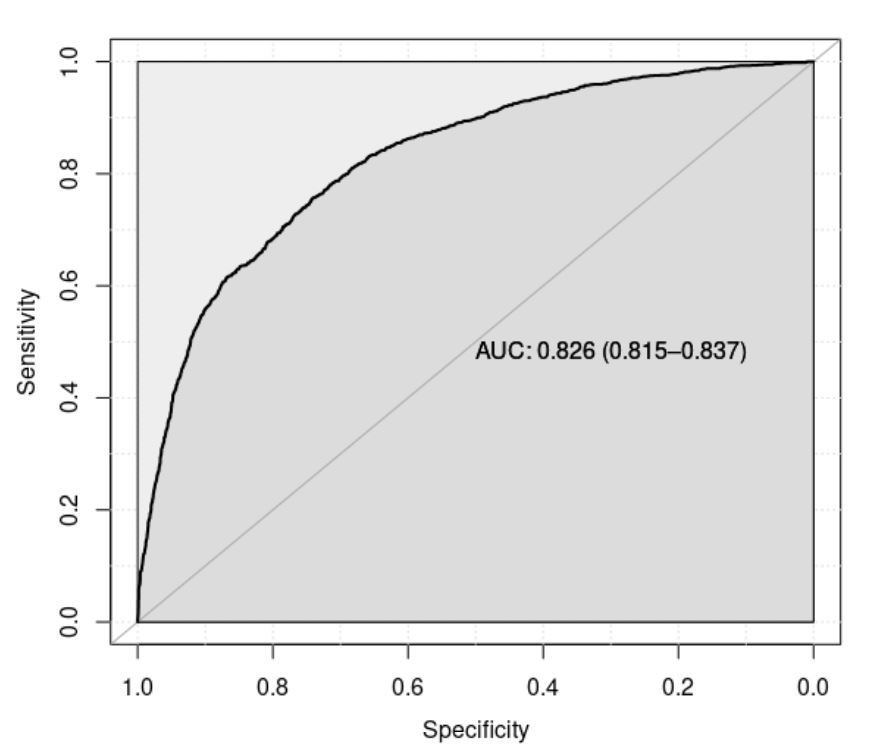

Supplement: S1 Fig — (TIF) [file pone.0252903.s001.tif]
