# Supplementary material for: External validation of a claims-based model to predict left ventricular ejection fraction class in patients with heart failure
Source: PLoS One. 2021 Jun 4;16(6):e0252903. doi: 10.1371/journal.pone.0252903 (PMC8177622; doi:10.1371/journal.pone.0252903)
Supplement: S1 Table — (PDF) [file pone.0252903.s002.pdf]

S1 Table. Operational definitions for the variables included in the EF class prediction algorithm

Variable	Variable name in SAS algorithm	ICD-9 codes Definition (Codes are ICD-9 diagnosis unless otherwise specified, all medical claims, inpatient and outpatient, should be used to define the conditions unless otherwise specified)	Corresponding ICD-10 Diagnosis / Procedure codes, or prescription codes	When measured
Gender	Male	N/A	N/A	On index date
Age	Age	N/A	N/A	On index date
Systolic heart failure	hf_systolic	ICD-9 code of 428.2x (not co-occurring with 428.3x)	I50.2x (not co-occurring with I50.3x)	On index date
Diastolic heart failure	hf_diastolic	a code of 428.3x (not co-occurring with 428.2x)	I50.3x (not co-occurring with I50.2x)	On index date
Left heart failure	hf_left	a code of 428.1x (not co-occurring with more specific systolic or diastolic HF codes of 428.2x or 428.3x)	I50.1 (not co-occurring with more specific systolic or diastolic HF codes of I50.2x or I50.3x)	On index date
Unspecified heart failure	hf_unspecified	codes of 428.0x, 428.4x, 428.9x as well as instances of recording of both 428.2x and 428.3x	codes of I50.4x, I50.8x, or I50.9 as well as instances of recording of both I50.2x and I50.3x	On index date
Index diagnosis recorded during an outpatient visit	index_dx_out	If index diagnosis occurs in an outpatient claim	N/A	On index date
Number of hospitalizations for CHF	hosp_chf	Count variable where CHF is the primary diagnosis in an inpatient stay	N/A	6 month prior to the index date (including index date)
Implantable cardioverter defibrillator	dx_defibrillator	V45.02 (ICD-9 diagnosis code)<br>37.94-37.98 (ICD-9 procedure codes)	Z95.810, [(02H60KZ, 02H63KZ, 02H64KZ, 02H70KZ, 02H73KZ, 02H74KZ, 02HK0KZ, 02HK3KZ, 02HK4KZ, 02HL0KZ, 02HL3KZ, 02HL4KZ) + (0JH608Z, 0JH638Z, 0JH808Z,	6 month prior to the index date to (index date+30 days)
Ace inhibitor	rx_ace	Benazepril, captopril, enalapril, fosinopril, lisinopril, moexipril, perindopril, quinapril, ramipril, trandolapril from prescription claims		6 month prior to the index date to (index date+30 days)
Mineralocorticoid receptor antagonist	rx_antagonist	Eplerenone, spironolactone from prescription claims		6 month prior to the index date to (index date+30 days)
Beta blocker	rx_bblocker	Acebutolol, atenolol, betaxolol, bisoprolol, carteolol, carvedilol, esmolol, labetalol, metoprolol, nadolol, nebivolol, penbutolol, pindolol, propranolol, timolol from prescription claims		6 month prior to the index date to (index date+30 days)
Digoxin	rx_digoxin	Digoxin from prescription claims		6 month prior to the index date to (index date+30 days)
Loop diuretic	rx_loop_diuretic	Bumetanide, furosemide, torsemide, ethacrynic acid from prescription claims		6 month prior to the index date to (index date+30 days)
Nitrate	rx_nitrates	Nitroglycerin, isosorbide dinitrate, isosorbide mononitrate, ranolazine from prescription claims		6 month prior to the index date to (index date+30 days)
Thiazide diuretic	rx_thiazide	Bendroflumethiazide, Benzthiazide, Chlorothiazide, Chlorthalidone, Hydrochlorothiazide, Indapamide, Methyclothiazide, Metolazone, Polythiazide, Quinethazone, Trichlormethiazide from prescription claims		6 month prior to the index date to (index date+30 days)
Atrial fibrillation	dx_afib	427.3x	I48.x	6 month prior to the index date to (index date+30 days)
Anemia	dx_anemia	280.xx	D50.x	6 month prior to the index date to (index date+30 days)
		281.xx	D51.0, D51.1, D51.3, D51.8, D52.0, D52.1, D52.8, D52.9, D53.0, D53.1, D53.8, D53.9	
		282.xx	D58.0, D58.1, D55.0, D55.1, D55.8, D56.x, D57.x, D58.2, D58.8, D58.9	
		283.xx	D59.x	
		284.xx	D61.x	
		285.xx	D50.x, D51.x, D52.x, D53.x, D55.x, D56.x, D57.x D58.x, D59.x, D60.x, D61.x, D62.x, D63.x, D64.x	
		ICD-9 Procedure		

Coronary artery bypass graft	dx_cabg	36.1x	0210093, 02100A3, 02100J3, 02100K3, 02100Z3, 0210493, 02104A3, 02104J3, 02104K3, 02104Z3, 021008W, 021009W, 02100AW, 02100JW, 02100KW, 021048W, 021049W, 02104AW, 02104JW, 02104KW, 021108W, 021109W, 02110AW, 02110JW, 02110KW, 021148W, 021149W, 02114AW, 02114JW, 02114KW, 021208W, 021209W, 02120AW, 02120JW, 02120KW, 021248W, 021249W, 02124AW, 02124JW, 02124KW, 021308W, 021309W, 02130AW, 02130JW, 02130KW, 021348W, 021349W, 02134AW, 02134JW, 02134KW, 0210088, 0210089, 021008C, 0210098, 0210099, 021009C, 02100A8, 02100A9, 02100AC, 02100J8, 02100J9, 02100JC, 02100K8, 02100K9, 02100KC, 02100Z8, 02100Z9, 02100ZC, 0210488, 0210489, 021048C, 0210498, 0210499, 021049C, 02104A8, 02104A9, 02104AC, 02104J8, 02104J9, 02104JC, 02104K8, 02104K9, 02104KC, 02104Z8, 02104Z9, 02104ZC,	6 month prior to the index date to (index date+30 days) 6 month prior to the index date to (index date+30 days)
			0211088, 0211089, 021108C, 0211098, 0211099, 021109C, 02110A8, 02110A9, 02110AC, 02110J8, 02110J9, 02110JC, 02110K8, 02110K9, 02110KC, 02110Z8, 02110Z9, 02110ZC, 0211488, 0211489, 021148C, 0211498, 0211499, 021149C, 02114A8, 02114A9, 02114AC, 02114J8, 02114J9, 02114JC, 02114K8, 02114K9, 02114KC, 02114Z8, 02114Z9, 02114ZC, 021208C, 021209C, 02120AC, 02120JC, 02120KC, 02120ZC, 021248C, 021249C, 02124AC, 02124JC, 02124KC, 02124ZC, 021308C, 021309C, 02130AC, 02130JC, 02130KC, 02130ZC, 021348C, 021349C, 02134AC, 02134JC, 02134KC, 02134ZC, 021008F, 021009F, 02100AF, 02100JF, 02100KF, 02100ZF, 021048F, 021049F, 02104AF, 02104JF, 02104KF, 02104ZF, 0210083, 0210093, 02100A3, 02100J3, 02100K3, 02100Z3, 0210483, 0210493, 02104A3, 02104J3, 02104K3, 02104Z3	
		36.2x	021K0Z8, 021K0Z9, 021K0ZC, 021K0ZW, 021K4Z8, 021K4Z9, 021K4ZC, 021K4ZW, 021L0Z8, 021L0Z9, 021L0ZC, 021L4Z8, 021L4Z9, 021L4ZC	
		CPT4:		
		33510 – 33536		
		33545		
		33572		
Cardiomyopathy	dx_cardiomyopathy	425.x	I42.x, I43.x	6 month prior to the index date to (index date+30 days)
COPD	dx_copd	491.xx	J41.x, J42.x, J44.x	6 month prior to the index date to (index date+30 days)
		492.xx	J43.x	
		496.xx	J44.9	
		493.2x	J44.0, J44.1, J44.9	
Depression	dx_depression	293.83	F06.30	6 month prior to the index date to (index date+30 days)
		296.2x	F32.x	
		296.3x	F33.x	
		296.9	F34.8, F39.x	
		298.0x	F32.3, F33.3	
		300.4x	F34.1	
		309.1x	F43.21	
		309.28	F43.23	
		311.xx	F32.9	
Hypertensive nephropathy	dx_htn_nephropathy	403.xx, 404.xx	I12.x, I13.x	6 month prior to the index date to (index date+30 days)
Hyperlipidemia	dx_hyperlipidemia	272.xx	E78.x	6 month prior to the index date to (index date+30 days)
Hypertension	dx_hypertension	401.xx, 402.xx, 405.xx	I10.x, I11.x I15.x, I12.0, I12.9, I13.0, I13.10, I13.11, I13.2	6 month prior to the index date to (index date+30 days)
Hypotension	dx_hypotension	458.xx	I95.x	6 month prior to the index date to (index date+30 days)
Myocardial infarction	dx_mi	410.xx	I21.01, I21.02, I21.09, I21.11, I21.19, I21.21, I21.29, I21.3, I21.4, I22.0, I22.1, I22.2, I22.8, I22.9	6 month prior to the index date to (index date+30 days)
Obesity	dx_obesity	278	E65.x, E66.x, E67.x, E.68.x	6 month prior to the index date to (index date+30 days)
		278.01	E66.01	
		V85.3x	Z68.3x	
		V85.4x	Z68.4x	
		CPT codes		
		43842', '43843', '43846', '43847', '43848', 'G0443', 'G0447’		
		Prescriptions of orlistat, sibutramine, phentermine, benzphetamine, phendimetrazine, diethylpropion		
Other dysrythmias	dx_oth_dysrhythmia	427.0x	I47.0, I47.1	6 month prior to the index date to (index date+30 days)
		427.1x	I47.2	
		427.2x	I47.9	
		427.4x	I49.01, I49.02	
		427.6x	I49.40, I49.1, I49.2, I49.3, I49.49	
		427.8x	I49.5, R00.1, I49.8	

		427.9x	I49.9	
		785.0x	R00.0	
Psychosis	dx_psychosis	290.8x, 290.9x, 295.xx, 297.xx, 298.xx, 299.xx, 780.1x	F03.90, F20.X, F22.X, F32.3, F33.3, F28.X, F44.89, F23.X, F29.X, F84.0, F84.3, F84.5, F84.8, F84.9, R44.0, R44.2, R44.3, R55.X, R56.00, R56.01, R56.1, R56.9, R42.X, G47.9, G47.30, G47.00, G47.10, G47.20, G47.8, F51.8, R50.2, R50.9, R50.81, R50.82, R50.83, R68.83, R68.0, R50.84, R53.82, R53.2, G93.3, R53.1, R53.81, R53.83, R61.X, R68.12, R68.11, R41.2, R41.3, R68.81, R45.83, R52.XX, R41.82, R45.84, R68.89, F24, F25.0, F25.1, F25.8, F25.9	6 month prior to the index date to (index date+30 days)
Rheumatic heart disease	dx_rheumatic_heart	393-398.x	I05.XX-I09.XX	6 month prior to the index date to (index date+30 days)
Sleep apnea	dx_sleep_apnea	327.2x	G47.3X	6 month prior to the index date to (index date+30 days)
		780.51	G47.30	
		780.53	G47.30	
		780.57	G47.30	
Stable angina	dx_stable_angina	413.xx	I20.1, I20.8, I20.9, I25.111, I25.118, I25.119, I25.701, I25.708, I25.709, I25.711, I25.718, I25.719, I25.721, I25.728, I25.729, I25.731, I25.738, I25.739, I25.751, I25.758, I25.759, I25.761, I25.768, I25.769, I25.791, I25.798, I25.799	6 month prior to the index date to (index date+30 days)
Valve disorder	dx_valve_disorder	394.x	I05.0, I05.1, I05.2, I05.8, I05.9	6 month prior to the index date to (index date+30 days)
		395.x	I06.0, I06.1, I06.2, I06.8, I06.9	
		396.x	I08.0, I08.8, I08.9	
		397.x	I07.1, I07.2, I07.8, I09.89, I09.1	
		398.9x	I09.9, I09.81, I09.89	
		V42.2	Z95.3	
		V43.3	Z95.2	
		OR		
		ICD-9 procedure code		
		35.1x	02QF0ZZ, 02QG0ZZ, 02QH0ZZ, 02QJ0ZZ, 027F04Z, 027F0DZ, 027F0ZZ, 02NF0ZZ, 02QF0ZZ, 027G04Z, 027G0DZ, 027G0ZZ, 02NG0ZZ, 02QG0ZZ, 02VG0ZZ, 027H04Z, 027H0DZ, 027H0ZZ, 02NH0ZZ, 02QH0ZZ,	
		35.2x	02RF07Z, 02RF08Z, 02RF0JZ, 02RF0KZ, 02RF47Z, 02RF48Z, 02RF4KZ, 02RF4JZ, 02RG07Z, 02RG08Z, 02RG0KZ, 02RG37Z, 02RG38Z, 02RG3KZ, 02RG47Z, 02RG48Z, 02RG4KZ, 02RG0JZ, 02RG3JZ, 02RG4JZ, 02RH07Z, 02RH08Z, 02RH0KZ, 02RH47Z, 02RH48Z, 02RH4KZ, 02RH0JZ, 02RH4JZ, 02RJ07Z, 02RJ08Z, 02RJ0KZ, 02RJ37Z, 02RJ38Z, 02RJ3KZ, 02RJ47Z, 02RJ48Z, 02RJ4KZ, 02RJ0JZ, 02RJ3JZ, 02RJ4JZ	
		OR		
		one of the following CPT codes:		
		33660-33665		
		33400-33403		
		33420-33430		
		33460		
		33463-33468		
		33475		
		33496		
		0257T		
		0258T		
		0259T		
		0262T		
