# Supplementary material for: External validation of a claims-based model to predict left ventricular ejection fraction class in patients with heart failure
Source: PLoS One. 2021 Jun 4;16(6):e0252903. doi: 10.1371/journal.pone.0252903 (PMC8177622; doi:10.1371/journal.pone.0252903)
Supplement: S2 Table — (PDF) [file pone.0252903.s003.pdf]

S2 Table. Baseline Characteristics of HF Patients correctly and incorrectly classified by algorithm compared to Gold standard classification.

Variable	Correctly classified rEF cases (N=547)	rEF cases incorrectly classified as pEF (N=1,153)	Gold standard HFrEF (N=1,700)	Correctly classified pEF cases (N=5,094)	pEF cases incorrectly classified as rEF (N=207)	Gold standard HFpEF (N=5,301)
	N (%)	N (%)	N (%)	N (%)	N (%)	N (%)
Mean LVEF, (SD)	0.29 (0.09)	0.33 (0.08)	0.32 (0.09)	0.59 (0.07)	0.53 (0.07)	0.59 (0.07)
Demographics						
Male	401 (73.31)	751 (65.13)	1152 (67.76)	2542 (49.90)	145 (70.05)	2687 (50.69)
Age, mean (SD)	64.3 (13.7)	71.6 (13.5)	69.2 (14.0)	70.9 (13.5)	62.4 (15.8)	70.6 (13.7)
HF-related variables						
HF-specific ICD-9 and ICD-10 codes						
Systolic HF	398 (72.76)	259 (22.46)	657 (38.65)	343 (6.73)	133 (64.25)	476 (8.98)
Diastolic HF	-	83 (7.20)	83 (4.88)	1358 (26.66)	2 (0.97)	1360 (25.66)
Left HF	49 (8.96)	45 (3.90)	94 (5.53)	202 (3.97)	37 (17.87)	239 (4.51)
Unspecified HF	77 (14.08)	713 (61.84)	790 (46.47)	2906 (57.05)	24 (11.59)	2930 (55.27)
HF Hospitalizations, mean (SD)	0.22 (0.42)	0.14 (0.34)	0.16 (0.37)	0.08 (0.27)	0.09 (0.28)	0.08 (0.27)
Implantable cardioverter-defibrillator	112 (20.48)	133 (11.54)	245 (14.41)	76 (1.49)	35 (16.91)	111 (2.09)
HF diagnosis identified in outpatient claims	246 (44.97)	640 (55.51)	886 (52.12)	3032 (59.52)	114 (55.07)	3146 (59.35)
HF-related medication use			-			-
ACE inhibitors	363 (66.36)	605 (52.47)	968 (56.94)	1987 (39.01)	121 (58.45)	2108 (39.77)
Mineralocorticoid receptor antagonists	186 (34.00)	203 (17.61)	389 (22.88)	433 (8.50)	34 (16.43)	467 (8.81)
Beta blockers	345 (63.07)	653 (56.63)	998 (58.71)	2446 (48.02)	141 (68.12)	2587 (48.80)
Digoxin	36 (6.58)	65 (5.64)	101 (5.94)	111 (2.18)	7 (3.38)	118 (2.23)
Loop diuretics	323 (59.05)	629 (54.55)	952 (56.00)	2408 (47.27)	81 (39.13)	2489 (46.95)
Nitrates	115 (21.02)	170 (14.74)	285 (16.76)	480 (9.42)	39 (18.84)	519 (9.79)
Thiazide diuretics	238 (43.51)	391 (33.91)	629 (37.00)	1524 (29.92)	57 (27.54)	1581 (29.82)
Comorbidities						
Atrial fibrillation or flutter	204 (37.29)	519 (45.01)	723 (42.53)	1878 (36.87)	78 (37.68)	1956 (36.90)
Anemia	138 (25.23)	445 (38.59)	583 (34.29)	2057 (40.38)	64 (30.92)	2121 (40.01)
Coronary artery bypass graft	36 (6.58)	96 (8.33)	132 (7.76)	283 (5.56)	9 (4.35)	292 (5.51)
Cardiomyopathy	467 (85.37)	322 (27.93)	789 (46.41)	404 (7.93)	168 (81.16)	572 (10.79)
Chronic obstructive pulmonary disease	126 (23.03)	296 (25.67)	422 (24.82)	1497 (29.39)	42 (20.29)	1539 (29.03)
Depression	60 (10.97)	149 (12.92)	209 (12.29)	909 (17.84)	32 (15.46)	941 (17.75)
Hypertensive nephropathy	61 (11.15)	180 (15.61)	241 (14.18)	744 (14.61)	28 (13.53)	772 (14.56)
Hyperlipidemia	312 (57.04)	751 (65.13)	1063 (62.53)	3234 (63.49)	122 (58.94)	3356 (63.31)
Hypertension	413 (75.50)	952 (82.57)	1365 (80.29)	4225 (82.94)	150 (72.46)	4375 (82.53)
Hypotension	97 (17.73)	196 (17.00)	293 (17.24)	781 (15.33)	30 (14.49)	811 (15.30)
Myocardial infarction	189 (34.55)	247 (21.42)	436 (25.65)	531 (10.42)	77 (37.20)	608 (11.47)
Obesity	113 (20.66)	211 (18.30)	324 (19.06)	1228 (24.11)	49 (23.67)	1277 (24.09)
Other dysrhythmias	365 (66.73)	637 (55.25)	1002 (58.94)	2345 (46.03)	124 (59.90)	2469 (46.58)
Psychosis	165 (30.16)	374 (32.44)	539 (31.71)	1896 (37.22)	68 (32.85)	1964 (37.05)
Rheumatic heart disease	67 (12.25)	193 (16.74)	260 (15.29)	960 (18.85)	34 (16.43)	994 (18.75)
Sleep apnea	80 (14.63)	155 (13.44)	235 (13.82)	920 (18.06)	30 (14.49)	950 (17.92)
Stable angina	76 (13.89)	139 (12.06)	215 (12.65)	518 (10.17)	22 (10.63)	540 (10.19)
Valve disorders	69 (12.61)	209 (18.13)	278 (16.35)	1113 (21.85)	35 (16.91)	1148 (21.66)
